# Calix[n]arene/Pillar[n]arene-Functionalized Graphene Nanocomposites and Their Applications

**DOI:** 10.3389/fchem.2020.00504

**Published:** 2020-06-12

**Authors:** Qunpeng Duan, Lijie Wang, Fei Wang, Hongsong Zhang, Kui Lu

**Affiliations:** ^1^School of Materials and Chemical Engineering, Henan University of Engineering, Zhengzhou, China; ^2^School of Chemical Engineering and Food Science, Zhengzhou Institute of Technology, Zhengzhou, China

**Keywords:** calix[n]arene, pillar[n]arene, graphene, nanocomposites, functionalization

## Abstract

Calix[n]arenes and pillar[n]arenes, which contain repeating units of phenol and methane, are class of synthetic cyclic supramolecules. Their rigid structure, tunable cavity size, flexible functionalization, and rich host-guest properties make them ideal surface modifiers to construct functional hybrid materials. Introduction of the calix[n]arene/pillar[n]arene species to the graphene may bring new interesting or enhanced physicochemical/biological properties by combining their individual characteristics. Reported methods for the surface modification of graphene with calix[n]arene/pillar[n]arene utilize either covalent or non-covalent approaches. This mini-review presents the recent advancements in the functionalization of graphene nanomaterials with calix[n]arene/pillar[n]arene and their applications. At the end, the future outlook and challenges for the continued research of calix[n]arene/pillar[n]arene-functionalized graphene nanohybrids in the development of applied nanoscience are thoroughly discussed.

## Introduction

In recent years, as a two-dimensional sp^2^-hybridized carbon nanomaterial, graphene has attracted intense scientific interest since its discovery in 2004. It is described as the World's “thinnest” material, and it presents fascinating mechanical, electronic, thermal, optical, and chemical properties that have made it a promising material for potential use in various scientific fields such as nanoelectronics (Son et al., 2006), supercapacitors (Maiti et al., 2014), batteries (Takamura et al., 2007), sensors (Shao et al., 2010), and nanocomposites (Watcharotone et al., 2007). The development of these applications requires preservation of the single-layer of graphene in common solvents. However, it is difficult to construct single-layer graphene at ambient temperature. Graphene sheets tend to form irreversible agglomerates due to the attractive van der Waals forces between the graphene sheets or even re-aggregate through those same attractive forces if the sheets are not well-separated from each other (Li et al., 2008; Shan et al., 2009). Aggregation can be greatly reduced by attachment of other functionality to the graphene sheets. Such functionality should bring new properties, produce some desirable effects, and open up a new avenue to further utilize the resulting nanostructure as novel composite materials. One of the most promising strategies for the creation of functionalized graphene is using water-soluble supramolecules capable of

forming host-guest complexes with the target substrate (Guo et al., 2010; Kasprzak and Poplawska, 2018). The introduction of water-soluble supramolecules as functional molecules can effectively disperse graphene, and further introduce new or enhanced functions through combining their individual characteristics.

**Figure 1:**
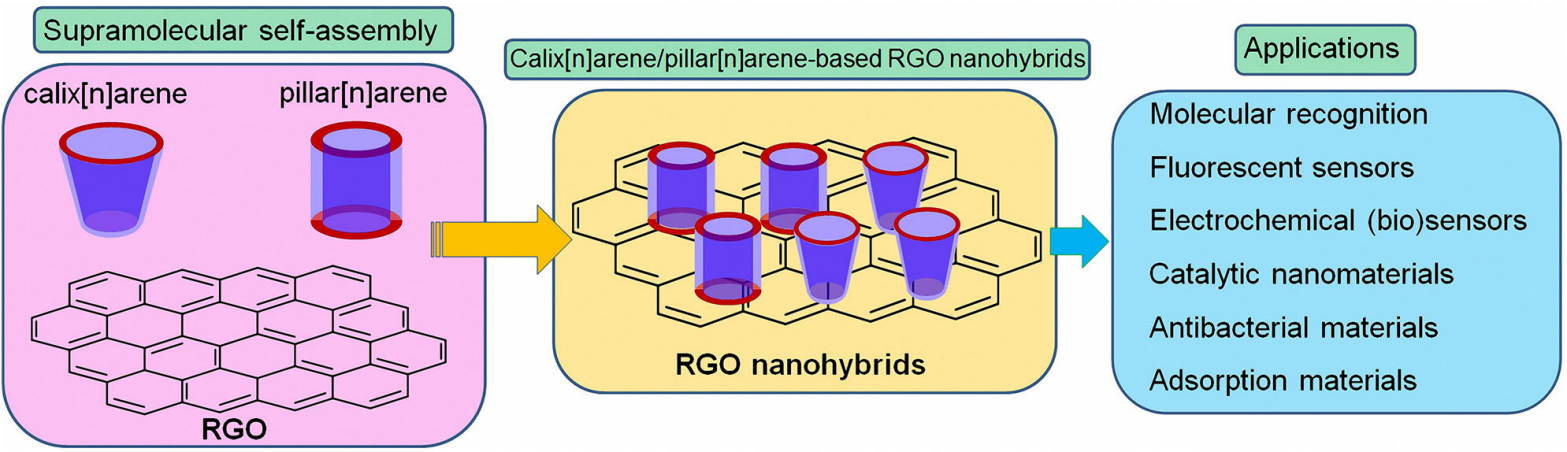
Calix[n]arene/pillar[n]arene-functionalized graphene nanohybrids and their applications.

**Figure 2:**
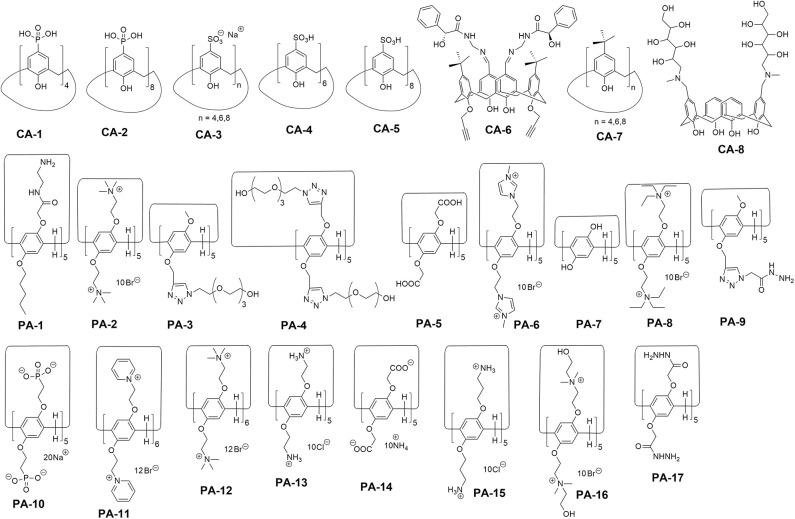
Structures of calix[n]arenes and pillar[n]arenes.

Calix[n]arenes and pillar[n]arenes are class of synthetic cyclic oligomers which contain repeating phenolic units and methane. Calix[n]arenes are generally known as the third class of supramolecules (Gutsche, 1998). Most of the supramolecular self-assembly and substrate-recognition patterns were first realized with calix[n]arenes and thereafter so were other supramolecules. Ease functionalization of both the upper and lower rims of calix[n]arenes have made them attractive versatile building blocks for supramolecular hybrid materials. Emerging as a relatively new class of calix[n]arene analogs, pillar[n]arenes and their derivatives have attracted particular interest in supramolecular chemistry and materials science during past decades (Ogoshi et al., 2008; Ogoshi, 2015; Wang et al., 2018a,b). Different from conventional calix[n]arenes, their symmetrical rigid pillar-shaped structures, tunable cavity size, easy functionalization, and unique host-guest recognition abilities inspire researchers to construct pillar[n]arene-based functional nanocomposite materials. In the present mini-review, we summarize the recent achievements in the design and application of graphene functionalized with calix[n]arene/pillar[n]arene (Figure 1). The structures of calix[n]arenes and pillar[n]arenes utilized to construct graphene hybrids are shown in Figure 2. Besides, the potential and challenges of the study of calix[n]arene/pillar[n]arene-functionalized graphene nanomaterials are summarized.

## Functionalization of Graphene With Calix[n]arenes

As the third-generation host molecules after crown ethers and cyclodextrins, calix[n]arenes have attracted continuous interest since they can form stable host-guest complexes with various guest molecules (Diamond and McKervey, 1996; Gutsche, 1998). It is noteworthy that calix[n]arenes can be used as modifiers to form calix[n]arenes-functionalized graphene composites by π-π stacking interactions and hydrogen-bonding interactions (Eroglu et al., 2013; Zhou et al., 2013a). The advantage of water-soluble calix[n]arenes, particularly, *p*-phosphonate and *p*-sulfonated derivatives, is that it brings graphene high water-solubility and guest molecules trapped into calix[n]arenes are easily accessible to graphene.

*p*-Phosphonic acid calix[n]arene, an amphiphilic supramolecule with the phosphonate groups and hydroxide groups on the upper and lower rim, respectively, has been employed as a versatile stabilizing surfactant to effectively stabilize graphenes in water. In 2011, Zou et al. (2011) successfully prepared the first example of *p*-phosphonic acid calix[4]arene (**CA-1**) modified graphene. **CA-1** gives high stability to graphene in water. This **CA-1** modified graphene (**CA-1**@graphene) can be utilized as a highly effective template on nucleate ultra-small palladium nanoparticles (Pd NPs), which in turn stabilizes graphene. High-density 2D nano-arrays of platinum nanoparticles (Pt NPs) were achieved by using Pd-**CA-1**@graphene as galvanic reaction templates. Subsequently, Chen et al. (2013a,b); Chen et al. (2014) reported non-covalent functionalization of reduced graphene oxide (RGO) in an aqueous medium with *p*-phosphonic acid calix[8]arene (**CA-2**). Employing a simple and versatile drop-casting technique on interdigital electrodes, electrocatalytic ternary Pd/Pt/Ru-**CA-2**@graphene nanocomposite has been incorporated into a device for effective hydrogen sensing, which provides an attractive perspective in replacing traditional graphene oxide (GO) or RGO as an effective supporting material for noble metal nanostructures in energy storage, sensors, catalysis, and devices. In 2013, Eroglu et al. (2013) also constructed **CA-2** stabilized graphene for reversible nitrate uptake from aquatic effluents.

In supramolecular chemistry, fragments of sulfonic acids are usually employed in order to increase water solubility, which gives surficial active properties based on acid-base or ionic interactions. A significant contribution in the preparation and application of sulfonatocalix[n]arene-stabilized graphene has been made by many research groups. In 2013, Zhou et al. (2013a) reported that three kinds of *p*-sulfonatocalix[4,6,8]arenes sodium (**CA-3**) were successfully loaded onto RGO surface by utilizing a simple wet-chemical method. Significantly, **CA-3**@RGO-modified glassy carbon electrode (GCE) exhibited an enhanced electrochemical response to dye molecules and biomolecules due to the high supramolecular recognition and enrichment effect between **CA-3** and the biological and organic dye molecules. Subsequently, Yang et al. (2016b) developed an electrochemical sensing platform based on **CA-4** functionalized reduced graphene oxide (**CA-4**@RGO). Based on competitive host-guest recognition between **CA-4** and methylene blue/ cholesterol, **CA-4**@RGO-modified GCE was used as an electrochemical sensor for the efficient and selective detection of cholesterol without any enzyme or antibody. Later in 2018, Song et al. (2018) constructed a **CA-4**@RGO-modified gold electrode for the sensitive and selective detection of caspase-3. **CA-4**@RGO can be attached onto the gold electrode surface through host-guest recognition. Owing to the host-guest recognition of **CA-4**, electrical signal molecules methylene blue has more binding sites on the **CA-4**@RGO surface, which resulted in a higher electrochemical response to caspase-3.

In the meantime, Yang et al. (2015, 2016a) also constructed two simple and convenient fluorescence sensing platforms based on **CA-4** and **CA-5** functionalized reduced graphene oxide (**CA-4**@RGO and **CA-5**@RGO). Based on competitive host-guest recognition, **CA-4**@RGO and **CA-5**@RGO were used as a fluorescent probe for the sensitive and selective detection of tadalafil and aconitine, respectively. Later in 2016, Ye et al. (2016) also reported a facile competitive fluorescent sensing platform by using **CA-4**-MnO_2_@RGO composite as a receptor. The ternary nanocomposites **CA-4**-MnO_2_@RGO simultaneously possess the excellent quenching performance of MnO_2_@RGO and the high supramolecular recognition capability of **CA-4**, which was used as a fluorescent probe for the sensitive and selective detection of labetalol in human serum samples. Later in 2016, **CA-4** was also used as an effective particle stabilizer to create silver-graphene nanocomposites with enhanced antibacterial activity (Kellici et al., 2016).

In 2015, Mao et al. (2015) designed and synthesized a chiral *R*-mandelic acid calix[4]arene (**CA-6**). **CA-6** was successfully covalently grafted to GO through a click reaction to obtain **CA-6** modified graphene (**CA-6**@G). By taking advantages of both the functional calixarene and graphene, **CA-6**@G was used as a chiral probe for the highly sensitive and selective sensing of amino propanol enantiomers in a serum sample at the nM levels. Later in 2016, Zhang et al. (2016) reported that three kinds of calix[4,6,8]arenes (**CA-7**) were covalently anchored onto the surface of GO by esterification and polymerization of **CA-7** onto GO surfaces. Ongoing research confirms that the introduction of **CA-7** onto the GO surface facilitates the adsorption capacity toward neodymium ions. In 2018, Nurerk et al. (2018) prepared calix[4]arene-functionalized GO via covalent attachment of 4-tert-butylcalix[4]arene [**CA-7** (*n* = 4)] and GO. Subsequently, the prepared calix[4]arene@GO was absorbed and entrapped in porous polydopamine-coated cellulose acetate fiber (PDA-CFs) to fabricated calix[4]arene@GO/PDA-CFs. The developed calix[4]arene@GO/PDA-CFs was successfully applied as an adsorbent for analyzing aflatoxins from corn samples. Later in 2019, Nodeh et al. (2019) synthesized magnetic graphene on basis of *N*-methyl-*D*-glucamine functionalized calix[4]arene (**CA-8**) nanocomposite (**CA-8**@MGO) by covalent attachment of *N*-methyl-*D*-glucamine calix[4]arene (**CA-8**) and Fe_3_O_4_-graphene oxide (MGO). The synthesized **CA-8**@MGO was successfully utilized as an effective absorbent for the removal of chlorpyrifos and hexaconazole pesticides from water samples.

## Functionalization of Graphene With Pillar[n]arenes

Pillar[n]arenes are a relatively new class of macrocyclic hosts, which was first reported in 2008 (Ogoshi et al., 2008). These macrocyclic hosts have attracted much attention due to their synthetic accessibility and pillar-shaped three-dimensional structures. Practically, a series of pillar[n]arenes with good water solubility and recognition capability have been exploited to construct graphene hybrid materials to improve their water stability and dispersity, as well as to enhance their supramolecular recognition capability in many applications, including sensors, luminescence, electrocatalysis, and electronics, and therefore attracted wide research interest.

Zhou et al. (2013b) reported the first example of non-covalent functionalization of RGO with an amphiphilic pillar[5]arene (**PA-1**). The resulting functionalized graphene nanocomposite (**PA-1**@RGO) exhibits good water dispersibility. It also possesses excellent selective supramolecular recognition and enrichment capability toward guest molecules. In order to obtain ternary nanocomposites **PA-1**@RGO-AuNPs, gold nanoparticles (AuNPs) were self-assembled onto the surface of **PA-1**@RGO. Furthermore, these ternary nanocomposites **PA-1**@RGO-AuNPs exhibit enhanced electrochemical performance due to the synergistic effects of AP5 supramolecular recognition capability, high catalysis of AuNPs and excellent electron transport performances of RGO. Later in 2019, based on cationic pillar [5]arene (**PA-2**) modified RGO and PtPd bimetallic nanoparticles (PtPd NPs), Liang et al. (2019) also reported ternary nanocomposites **PA-2**@RGO-PtPd. To obtain these ternary nanocomposites, **PA-2**-decorated RGO was firstly prepared and then loaded with PtPd NPs. Glassy carbon electrode modified with **PA-2**@RGO-PtPd was employed for BPA detection with enhanced electrochemical performance, which is also caused by synergistic influences of **PA-2** host-guest recognition performance, high catalysis of PtPd NPs and conductivity of RGO.

In 2014, supramolecular functionalized graphene hybrid materials with conjugated tadpole-like (**PA-3**) and bola-amphiphilic pillar[5]arenes (**PA-4**) have been successfully prepared by Zhang et al. (2014). The strong interfacial adhesion between these pillar[5]arenes and graphene oxide was attributed to the strong hydrogen-bonding interactions. Ongoing research shows that these functionalized graphene hybrid materials have excellent biocompatibility and low cytotoxicity, which was used for *in vitro* dual-mode Raman and fluorescence imaging.

In 2015, Zhou et al. (2015) designed a sensing platform by the functionalization of RGO with a water-soluble carboxylated pillar[5]arene (**PA-5**) via covalent bonds. The resulting functionalized graphene exhibits good water dispersibility and enhanced fluorescence-quenching resistance as compared with native RGO. This fabricated sensing platform opens up prospects to sense and detect organic dye molecules and pesticides in aqueous solution.

Later in 2015, Chen's group investigated the role of cationic water-soluble pillar[5]arene (**PA-2**) containing 10 trimethylammonium groups on the microstructure of MoS_2_/RGO composites. They found that the modification of pillar[5]arene onto the surface of graphene oxide sheets (GOS) enhances the lithium storage performance of MoS_2_/RGO composites (Ye et al., 2015). A later study from the same research group showed that the functionalization of N-methylimidazole water-soluble pillar[5]arene (**PA-6**) on the GOS surface also enhances the lithium storage performance of MoS_2_/RGO composites (Yu et al., 2017).

In 2016, Liu et al. (2016) reported the preparation of graphene-based hybrid material modified with per-hydroxylated pillar[5]arene (**PA-7**) via covalent bonds and its electrochemical behavior. The resulting functionalized graphene modified electrode exhibits high sensitivity and selectivity to dopamine. This sensing platform opens an attractive perspective in practical sensing and detection of dopamine in biological samples. Tan et al. (2019a) later also developed an ultrasensitive electrochemical sensor for detecting methyl parathion based on cationic water-soluble pillar[5]arene (**PA-8**) modified RGO. This sensor has been utilized to determine methyl parathion in wastewater and soil samples with satisfactory results.

In 2016, Mao et al. (2016) developed a new strategy of introducing hydrazine-pillar[5]arene (**PA-9**) onto biocompatible graphene via covalent bonds. The resulting functionalized graphene can be used as an effective fluorescence sensing platform for the detection of paraquat both in living cells and mice with low cellular toxicity. This method provides an attractive perspective in developing a new methodology for quantifying intracellular imaging in live cells.

In 2017, Zhao et al. (2017) designed a fluorescence sensing platform for the detection of acetaminophen (AP) by utilizing amphiphilic pillar[5]arene functionalized reduced graphene oxide (**PA-1**@RGO) as a sensor. Because of the host-guest interaction, both acetaminophen and acridine orange can thread into the hydrophobic inner cavity of **PA-1** to form 1:1 inclusion complex with **PA-1**. The obtained **PA-1**@RGO can detect acetaminophen by the host-guest competition due to the stronger interaction between AP and **PA-1**. Subsequently, Tan et al. (2019b,c) reported two selective and sensitive fluorescence sensing platforms based on phosphate pillar[5]arene (**PA-10**) functionalized reduced graphene oxide (**PA-10**@RGO) and pyridinium pillar[6]arene (**PA-11**) modified reduced graphene oxide (**PA-11**@RGO). Based on competitive host-guest recognition, **PA-10**@RGO and **PA-11**@RGO were used as a fluorescent probe for sensitively detecting paraquat and 2,4,6-Trinitrophenol, respectively. At the same time, Tan et al. (2019d) also reported a fluorescent sensing platform for the detection of insulin based on fluorescence resonance energy transfer through competitive supramolecular recognition between cationic pillar[6]arene (**PA-12**) functionalized reduced graphene oxide (**PA-12**@RGO) and probe/insulin molecules. Rhodamine B, whose fluorescence is quenched by RGO-based fluorescence resonance energy transfer, was used as a probe molecule. When insulin was added into **PA-12**@RGO, Rhodamine B was displaced by insulin and an inclusion complex between **PA-12**@RGO and insulin was formed, which resulted in a “turn-on” fluorescent signal. The constructed fluorescent sensing platform has been successfully used to detect insulin in artificial serum.

Later in 2018, Yang's group developed a rapid and convenient electrochemical method for selectively recognizing tryptophan isomers (L-/D-Trp) based on the co-assembly of water-soluble cationic pillar[5]arene (**PA-13**) and anionic pillar[5]arene (**PA-14**) on the carboxylic graphene (C-Gra) modified electrode (Zhao et al., 2018). Differential pulse voltammetry results show that the peak currents of tryptophan isomers decrease with the increasing of the assembled pillar[5]arene layer number, whereas the difference in the value of the peak current between L-Trp and D-Trp increase with the increased layers, demonstrating an effective route for the immobilization of pillar[5]arene for discriminating the tryptophan isomers. The same research group later reported the same co-assembly of water-soluble cationic pillar[5]arene (**PA-15**) and anionic pillar[5]arene (**PA-14**) on the C-Gra-modified electrode for the selective recognition of nitrophenol isomers (Tan et al., 2018).

In 2019, Sun et al. (2019) reported that silver nanoparticles (AgNPs) functionalized by carboxylated pillar[5]arene (**PA-5**) could be immobilized on GO surface by hydrogen bond and π-π stacking interaction between **PA-5** molecules and GO. GCE modified with **PA-5**@AgNPs-GO hybrid material demonstrates pronounced sensitivity toward paraquat, with limit detection for paraquat of 1.0 × 10^−8^ M. Such excellent performance can be explained by the synergic electrocatalytic effect of AgNPs and GO, and the host-guest recognition of **PA-5** for paraquat. Hou et al. (2019) later successfully prepared a simple and sensitive electrochemical sensor, in which hydroxylatopillar[5]arene (**PA-16**) stabilized AuNPs were anchored on electrochemically reduced graphene oxide (ERGO) by π-π stacking interaction between **PA-16** molecules and ERGO. The obtained hybrid composites **PA-16**@AuNPs-ERGO modified electrode show excellent detection sensitivity toward methyl parathion due to the combined advantages of high catalysis of AuNPs, **PA-16** host-guest recognition performance and conductivity of ERGO.

In 2020, Guo et al. (2020) developed a simple strategy for one-pot preparation of hydrazide-pillar[5]arene (**PA-17**) modified RGO (**PA-17**@RGO) hybrid material by the redox reaction between GO and **PA-17** in aqueous solution, in which **PA-17** served as the reducing agent and stabilizer. In the whole process, there was no need to add additional reducing agents. The few-layer **PA-17**@RGO composites constructed exhibited good water dispersibility and stability. The **PA-17**@RGO composite as electrode material displayed a high initial specific capacitance and great rate capability due to the synergistic effect of RGO and **PA-17**. This study provides an attractive perspective in expanding the development of supramolecular composites in energy storage.

## Conclusion and Outlook

In this mini-review, we overviewed recent progress in functionalization and applications of graphene nanomaterials with calix[n]arene/pillar[n]arene. New interesting approaches for constructing calix[n]arene/pillar[n]arene-functionalized graphene nanocomposites are reported. Bridging the supramolecular host-guest properties of calix[n]arene/pillar[n]arene hosts and the unique properties of graphene still inspires the scientific community to generate various important findings. However, some challenges remain in this emerging research field. First, the separation of excess calix[n]arene/pillar[n]arene modifiers from the functionalized graphene is a major concern. Therefore, special consideration should be given to the appropriate design of the modification protocol. Second, low-cost, efficient, and environmentally friendly approaches have to be developed for the preparation of calix[n]arene/pillar[n]arene-functionalized graphene sheets in large quantities with controllable sizes, layer thickness, compositions, and defects. Third, the properties and functions of the calix[n]arene/pillar[n]arene-functionalized graphene nanocomposites depend strongly on their microstructures. Therefore, in order to synthesize calix[n]arene/pillar[n]arene-functionalized graphene nanocomposites with desirable nanostructured architectures, the assembly behaviors of graphene with calix[n]arene/pillar[n]arene require to be studied more clearly. Finally, the applications of calix[n]arene/pillar[n]arene-functionalized graphene nanocomposites are at their initial stages. They need to be investigated systematically from both experimental and theoretical aspects. Altogether, we believe the combination of graphene materials with calix[n]arene/pillar[n]arene will produce a series of advanced materials with highly specific functionalities and potential applications having as the limit only the imagination.

## Author Contributions

QD supervised the project and mainly wrote the paper. All authors extensively reviewed the manuscript and approved the final version of the manuscript to be submitted.

## Conflict of Interest

The authors declare that the research was conducted in the absence of any commercial or financial relationships that could be construed as a potential conflict of interest.
